# Real-world experience of renal outcomes and management of PrEP users with estimated glomerular filtration rates less than 60 mL/min/1.73 m2

**DOI:** 10.1136/sextrans-2024-056411

**Published:** 2025-02-13

**Authors:** Victoria Tittle, Nicolo Girometti, Jonathan Joseph Goodfellow, Stephen Cole, Sheel Patel, Marta Boffito, Sheena McCormack, Rachael Jones

**Affiliations:** 1 56 Dean Street, Chelsea and Westminster Hospital NHS Foundation Trust, London, UK; 2 Department of HIV/GUM, Chelsea and Westminster Hospital NHS Foundation Trust, London, UK; 3 GenitoUrinary and HIV Medicine, Chelsea and Westminster Hospital NHS Foundation Trust, London, UK; 4 MRC CTU, University College London, London, UK

**Keywords:** RENAL, MANAGEMENT, PREP, HIV

## Abstract

This retrospective service evaluation reviews practical measures for pre-exposure prophylaxis (PrEP) users with estimated glomerular filtration rate (eGFR) <60 mL/min/1.73 m^2^. Of 35 724 PrEP users, 361 had an eGFR <60 mL/min/1.73 m^2^, of whom 16% required non-tenofovir disoproxil/emtricitabine PrEP formulations. Majority of cases required simple and conservative management, with repeat sampling and/or stopping of the supplements and/or recreational drugs.

## Introduction

 Pre-exposure prophylaxis (PrEP) for HIV prevention using tenofovir disoproxil/emtricitabine (TDx/FTC) has infrequent renal adverse events.^1^ Data on how to best manage PrEP users with renal impairment while taking TDx/FTC PrEP are limited. PrEP trials tend to recruit participants with a baseline creatinine clearance (CrCl) ≥60 mL/min, and in studies where individuals were observed to develop a CrCl <60 mL/min, individuals were advised to stop TDx/FTC until CrCl recovered, with 70% and 96% rebounding to >75% of baseline CrCl after 4 and 8 weeks of cessation, respectively.^2 3^ The British HIV Association and the British Association for Sexual Health and HIV guidelines recommend case-by-case decisions for those with an estimated glomerular filtration rate (eGFR) <60 mL/min/1.73 m^2^ with HIV acquisition risk.^4^

We previously reported 0.8% of our 2018–2019 PrEP cohort were reviewed for renal concerns and listed the outcomes of those who had eGFR <60 mL/min/1.73 m^2^.^5^ Other real-world PrEP settings also reported 1%–2% of cohorts have renal concerns, but with little or limited information on how cases were managed.^6 7^

In May 2023, tenofovir alafenamide/emtricitabine (TAF/FTC)-based PrEP became available on the National Health Service (NHS) in England for individuals in whom TDx/FTC is contraindicated, for example, for renal and bone indications or for those under 18 years of age. In October 2023, the European AIDS Clinical Society (EACS) noted that TAF/FTC could be considered, if available, where TDx/FTC was precluded, and that pharmacokinetic and pharmacodynamic (PK/PD) studies comparing TDx/FTC with TAF/FTC suggest the same starting and stopping rules can be followed for both drugs; TAF/FTC PrEP licensing only cites daily dosing.^8^ With the addition of TAF/FTC PrEP availability and the widening access to TDx/FTC via the NHS, we evaluated our service and present descriptive outcomes of those who presented to our clinical service with a baseline eGFR <60 mL/min/1.73 m^2^ and those who developed renal impairment on PrEP during follow-up, detailing case management.

## Methods

This is a retrospective case note review of clinical data from 1 January 2021 to 31 December 2022 from three sexual health services within Chelsea and Westminster NHS Trust, London (UK). Electronic patient records were interrogated to obtain demographics, sexual health histories, PrEP use, type of attendance (PrEP start or PrEP follow-up) and renal laboratory test outcomes for each PrEP attendance to identify PrEP users with at least one eGFR <60 mL/min/1.73 m^2^. PrEP users with at least one eGFR <60 mL/min/1.73 m^2^ had clinical note reviews to determine potential reasons and contributing factors for low eGFR. Case management, baseline eGFR values at the time of initial PrEP provision within the Trust and the most recent PrEP usage were collected. Demographics were categorised according to national reporting requirements for the UK Health Security Agency and the National Institute for Health and Care Excellence (NICE) chronic kidney disease (CKD) classification. Urine albumin to creatinine ratios were not available consistently as these are not routinely recommended in UK PrEP monitoring guidance.^9^ Data analysis was descriptive and performed on STATA V.17.0. To protect patient anonymity, data with fewer than five PrEP users are presented as <5, and with five to nine users presented as <10.

## Results

Of 35 724 PrEP users with at least one PrEP activity recorded, 361 (1%) had an eGFR <60 mL/min/1.73 m^2^ on at least one occasion. Of those with an eGFR <60 mL/min/1.73 m^2^ (n=361), the median age was 45 years old (IQR 37–56 years old), the median number of days between eGFR at PrEP start and latest eGFR recorded was 1015 days (IQR 546–1512 days), the median number of days from eGFR at PrEP start to having an eGFR <60 mL/min/1.73 m^2^ was 561 days (IQR 111–1064 days), and the median number of days from eGFR <60 mL/min/1.73 m^2^ to latest eGFR on record was 401 days (IQR 146–650 days).

Management and outcomes of those with an eGFR <60 mL/min/1.73 m^2^ are presented in table 1. Demographics, PrEP usage and renal medical history at PrEP start, linked with baseline renal function test results (eGFR), are provided in detail in online supplemental table 1, with causes and issues associated with an eGFR <60 mL/min/1.73 m^2^ in online supplemental table 2.

**Table 1:** Management and outcomes of patients with eGFR <60 mL/min/1.73 m^2^ with measures taken at the time of low eGFR reading and latest eGFR readings

Measures to manage patients		eGFR at the time of low eGFR testing			Outcome: most recent eGFR testing result					
		15–29	30–44	45–59	15–29	30–44	45–59	60–89	≥90	Total
Advised to repeat blood test	Advised to repeat blood test	<5	<5	97				73	26	99
	Advised to repeat blood test, but did not reattend		<5	<10						<10
	Advised to repeat blood test, with normal CG calculation			<5			<5			<5
Confirmed low eGFR (<60 mL/min/1.73 m^2^) and TAF/FTC PrEP advised with renal monitoring			<5	<5		<5	<5	<5		<10
Continued with previous daily TDx/FTC PrEP dosing				<5					<5	<5
Lost to follow-up			<5	18						19
Managed drug–drug interactions				<5				<5		<5
Managed infection				<5				<5	<5	<5
Optimised comorbidities	Optimised comorbidities			<5				<5		<5
	Optimised comorbidities and switched to EBD or TTSS TDx/FTC			<5				<5		<5
	Optimised comorbidities and switched to EBD or TTSS TDx/FTC (eGFR stable and known CKD)			<5			<5			<5
	Optimised comorbidities and switched to EBD or TTSS TDx/FTC and did not reattend			<5						<5
	Optimised comorbidities and switched to EBD or TTSS TDx/FTC, TAF/FTC advised			<5				<5		<5
	Optimised comorbidities but did not reattend			<5						<5
	Optimised comorbidities, TAF/FTC advised			<5			<5			<5
PrEP not started or stopped	PrEP not started (clinician decision)		<5							<5
	PrEP not started (patient choice)		<5	<10						<10
	PrEP stopped (by clinician)		<5	<5						<5
	PrEP stopped (by PrEP user)		<5	<5						<10
Protein and/or other supplements and/or recreational drugs	Reduced supplements and/or recreational drugs and repeat sample			84				63	21	84
	Reduced supplements and/or recreational drugs and repeat sample (eGFR stable)			<5			<5			<5
	Reduced supplements and/or recreational drugs and repeat sample and under investigation			<5			<5			<5
	Reduced supplements and/or recreational drugs and repeat sample but did not reattend		<5	12						14
	Reduced supplements and/or recreational drugs and repeat sample, with normal CG calculation			<5			<5			<5
EBD TDx/FTC dosing	Switched or continued EBD TDx/FTC		<5	22				22	<5	23
	Switched or continued EBD TDx/FTC and advised to stop supplements			<5				<5		<5
	Switched or continued EBD TDx/FTC and advised to stop supplements, TAF/FTC advised			<5			<5			<5
	Switched or continued EBD TDx/FTC and advised to stop supplements, with normal CG calculation			<5			<5			<5
	Switched or continued EBD TDx/FTC but did not reattend		<5	<5						<10
	Switched or continued EBD TDx/FTC, TAF/FTC advised			<5		<5	<5			<5
Switched to TAF/FTC	Switched to daily TAF/FTC		<10	45	<5	<5	22	25	<5	52
	Switched to EBD TAF/FTC PrEP			<10		<5	<5			<10
Switched to LA CAB		<5			<5					<5
Total		<5	25	334	<5	<10	41	197	52	

<5=under 5 users; <10=under 10 users.

CG, Cockroft-Gault; CKD, chronic kidney disease; EBD, event-based dosing (2,1,1); eGFR, estimated glomerular filtration rate; LA CAB, long-acting cabotegravir; PrEP, pre-exposure prophylaxis; TAF/FTC, tenofovir alafenamide/emtricitabine; TDx/FTC, tenofovir disoproxil/emtricitabine; TTSS, single TDx/FTC dose on Tuesday, Thursday, Saturday and Sunday.

## Discussion

About 1% of PrEP users were found to have a low eGFR (defined as <60 mL/min/1.73 m^2^) at least once during the 2-year evaluation period, reflecting other real-world experiences of renal monitoring among PrEP users and highlighting the small number of patients who require further renal intervention.^5^,^7^ This should reassure services expanding PrEP access, simplifying or developing novel methods of PrEP delivery.

Of those with a low eGFR, a third (n=109) had a baseline eGFR <60 mL/min/1.73 m^2^ when starting PrEP at our service, in keeping with other findings.^7^ It should be noted that our data could be an overestimate, as prior to NHS PrEP availability PrEP users could self-source medication online and this information was not systematically captured at PrEP initiation from our clinical service, adding to the limited data of PrEP initiations with a low baseline eGFR.

A third of cases (n=109) with low eGFR at baseline or on follow-up had contributing comorbidities. Comorbidity optimisation was vital for those with a cardiovascular disease history and diabetes. Blood pressure check and glycaemic screening were routinely performed within our ‘PrEP review’ service and required engagement with community practitioners to optimise care of chronic health conditions to support the continuation of TDx/FTC. The historical lack of communication between sexual health services and general practitioners in the UK is an issue and continues to grow as the number of PrEP users increases nationally and alternative PrEP options become available to those who were previously unable to take TDx.^5^ There were 28 cases of new renal pathologies that required specialist renal medicine input. Having accessible renal specialist services is essential for PrEP services who are planning substantial scale-up of PrEP provision.

Most cases with a low eGFR were found to have eGFR values between 45 mL/min/1.73 m^2^ and 59 mL/min/1.73 m^2^. Some (55%) were managed conservatively with repeat sampling with or without stopping protein supplements and/or recreational drugs. Many of those with low eGFR were taking supplements (40%); protein supplement intake varied from intermittent use to regular daily use, which would require a cessation of intake for a minimum of 2 days prior to repeating the sample.^5^ This may reflect a high proportion of protein supplement usage among the cohort at 56 Dean Street, where 94% of these cases were seen, compared with other services in this evaluation.^7^

Overall, 16% (n=58) of those with a low eGFR required an alternative formulation of PrEP; 57 PrEP users started TAF/FTC and 1 user on intramuscular long-acting cabotegravir (CAB), following discussion within our multidisciplinary PrEP service. Nearly half of the users who switched to daily TAF/FTC (n=27/52) saw eGFR improving to >60 mL/min/1.73 m^2^. One case had a borderline NICE CKD stage 3b–4 on starting TAF/FTC, and on their latest results showed CKD stage 4, but this is in keeping with the range of their results. Due to infrequent sexual activity, a number of PrEP users with known CKD did not wish to switch to daily TAF/FTC from event-based dosing (EBD) of TDx/FTC, given the lack of studies comparing these agents and data supporting 50% reduction of TDx/FTC from daily use in those with eGFR 50–60 mL/min/1.73 m^2^.^10^ Having discussed the PK/PD data of TAF/FTC PrEP, and referred to the EACS PrEP guidelines,^8^ five users eligible for TAF/FTC PrEP were advised to pursue EBD or one pill on Tuesday/Thursday/Saturday/Sunday dosing. All of these PrEP users were made aware of the lack of clinical trial data and licensing for TAF/FTC PrEP advising daily use. To our knowledge, this is the first experience of less than daily dosing of TAF/FTC for PrEP use and CAB use in those with low eGFRs.

The median number of days from low eGFR to latest eGFR was 401 days, which suggests patient engagement with PrEP despite renal issues, but 13% (n=48) were lost to follow-up despite being contacted. One case acquired HIV.

The retrospective and observational nature of the data represents a limitation in determining the exact cause of the low eGFR, partly because this can be multifactorial. PrEP start dates were not always accurate, as PrEP users may have started PrEP prior to attending our clinical service or started PrEP later than the date of dispensing. The population is predominately white GBMSM (Gay, bisexual or other men who have sex with men).

As a service evaluation, we were satisfied with clinical pathways in place to identify those with a low eGFR and to provide management plan and/or chase follow-up. Further work to monitor the long-term outcomes of those with a low eGFR is being considered.

## Conclusion

This is the largest service evaluation describing the management of PrEP cases with an eGFR <60 mL/min/1.73 m^2^, with practical interventions in managing renal impairment and PrEP use. Our data are reassuring; only 1% of this cohort required further clinical review for eGFR concerns, of whom only 16% required non-TDx/FTC PrEP formulations, demonstrating that standard-of-care TDx/FTC is appropriate for the vast majority of PrEP users.

## Supplementary material

10.1136/sextrans-2024-056411 online supplemental file 1
