# Supplementary material for: Real-world experience of renal outcomes and management of PrEP users with estimated glomerular filtration rates less than 60 mL/min/1.73 m2
Source: Sex Transm Infect. 2025 Feb 13;101(6):e056411. doi: 10.1136/sextrans-2024-056411 (PMC12421093; doi:10.1136/sextrans-2024-056411)
Supplement: online supplemental file 1 [file sextrans-101-6-s001.docx]

**Supplementary tables**

**Supplement table 1. Baseline eGFR at PrEP start within the local clinical service and PrEP user demographics, PrEP usage and renal medical history**

| PrEP user demographics, PrEP usage and renal medical history | | Total * *n* = 359*,* (% of total) | Baseline eGFR at PrEP start | |
| --- | --- | --- | --- | --- |
|  |  |  | eGFR ≥ 60 min/ml/1.73m2 *n* | eGFR < 60 min/ml/1.73m2 *n* |
| Age (years old) | 20-29 | 21 (6) | 14 | <10 |
|  | 30-39 | 95 (26) | 67 | 28 |
|  | 40-49 | 108 (30) | 73 | 35 |
|  | 50-59 | 72 (20) | 56 | 16 |
|  | ≥60 | 63 (18) | 40 | 23 |
| Ethnicity | White | 232 (65) | 170 | 62 |
|  | Mixed | 26 (7) | 15 | 11 |
|  | Asian | 18 (5) | 11 | <10 |
|  | Black | 50 (14) | 32 | 18 |
|  | Other | 33 (9) | 22 | 11 |
| Gender and sexual orientation | Cis-gendered men who have sex with men | 343 (95) | 240 | 103 |
|  | Transgender women | 13 (4) | <10 | <10 |
|  | Cis-gendered women | <5 (0) | <5 | . |
|  | Cis-gendered heterosexual men** | <5 (1) | <5 | . |
| Baseline eGFR stage (min/ml/1.73m2) | >90 | 54 (15) | 54 | . |
|  | 60-89 | 196 (55) | 196 | . |
|  | 45-59 | 97 (27) | . | 97 |
|  | 30-44 | 12 (3) | . | 12 |
| PrEP start regime | Daily TDx/FTC | 219 (61) | 167 | 52 |
|  | EBD TDx/FTC | 125 (35) | 83 | 42 |
|  | TAF/FTC | <5 (1) | 0 | <5 |
|  | LA CAB | <5 (0) | 0 | <5 |
|  | Didn’t start PrEP | 10 (3) | 0 | 10 |
| Known renal pathology at PrEP start | No | 277 (77) | 212 | 65 |
|  | Yes | 80 (22) | 38 | 42 |
|  | Unknown | <5 (1) | 0 | <5 |
| Developed new renal pathology | Yes | 28 (8) | 21 | <10 |
|  | Unknown | <5 (1) | <5 | <5 |
| Total |  |  | 250 | 109 |

<5 = under 5 users

<10 = under 10 users

* Baseline eGFR readings were missing for two PrEP users

**cis-gender heterosexual identifying men who had sex with men

**Supplementary table 2 – Issues raised from patient histories which may have been contributing factors for eGFR <60 ml/min/1.73m2 at time of test and breakdown according to NICE eGFR CKD stages**

| **Causes** | **Total, *n* (% on total of 387*****)** | **eGFR (ml/min/1.73m2) (n)** | | | **Total** |
| --- | --- | --- | --- | --- | --- |
|  |  | **15-29** | **30-44** | **45-59** |  |
| Under further investigation | <5 (1) |  |  | <5 | <5 |
| Relating to comorbidities | 109 (28) | <5 | 18 | 90 | 109 |
| Drug-Drug Interactions | <5 (1) |  |  | <5 | <5 |
| Possible dehydration | 15 (4) |  |  | 15 | 15 |
| New or known renal pathology at the time of eGFR ^ | <10 (2) |  | <5 | <10 | <10 |
| LTFUP (before a more detailed history could be obtained) | <10 (2) |  |  | <10 | <10 |
| Repeat result normalised (eGFR >60) with no other cause identified | 68 (18) | <5 | <5 | 66 | 68 |
| Concurrent infection (STI, MPox, UTI) | 11 (3) |  |  | 11 | 11 |
| Supplement related (including protein supplements) and/or recreational drugs | 144 (37) |  | <5 | 140 | 144 |
| Possible TDx related toxicity | 18 (5) |  | <5 | 16 | 18 |

<5 = under 5 users

<10 = under 10 users

*26 PrEP users had more than one cause recorded
^ A further 19 cases developed renal pathologies, but after further investigation
